# Atlas-based automatic segmentation of head and neck organs at risk and nodal target volumes: a clinical validation

**DOI:** 10.1186/1748-717X-8-154

**Published:** 2013-06-26

**Authors:** Jean-François Daisne, Andreas Blumhofer

**Affiliations:** 1 Radiation Oncology Dept, Clinique & Maternité Ste-Elisabeth, Place Louise Godin 15, 5000 – Namur, Belgium; 2 Brainlab AG, Kapellenstraße 12, 85622 - Feldkirchen, Germany

**Keywords:** Automatic segmentation, Head and neck cancer, Radiotherapy

## Abstract

**Background:**

Intensity modulated radiotherapy for head and neck cancer necessitates accurate definition of organs at risk (OAR) and clinical target volumes (CTV). This crucial step is time consuming and prone to inter- and intra-observer variations. Automatic segmentation by atlas deformable registration may help to reduce time and variations. We aim to test a new commercial atlas algorithm for automatic segmentation of OAR and CTV in both ideal and clinical conditions.

**Methods:**

The updated Brainlab automatic head and neck atlas segmentation was tested on 20 patients: 10 cN0-stages (ideal population) and 10 unselected N-stages (clinical population). Following manual delineation of OAR and CTV, automatic segmentation of the same set of structures was performed and afterwards manually corrected. Dice Similarity Coefficient (DSC), Average Surface Distance (ASD) and Maximal Surface Distance (MSD) were calculated for “manual to automatic” and “manual to corrected” volumes comparisons.

**Results:**

In both groups, automatic segmentation saved about 40% of the corresponding manual segmentation time. This effect was more pronounced for OAR than for CTV. The edition of the automatically obtained contours significantly improved DSC, ASD and MSD. Large distortions of normal anatomy or lack of iodine contrast were the limiting factors.

**Conclusions:**

The updated Brainlab atlas-based automatic segmentation tool for head and neck Cancer patients is timesaving but still necessitates review and corrections by an expert.

## **Background**

Over the past fifteen years, head and neck cancer (HNC) radiotherapy treatment has quickly shifted from classical two-dimensional (2D) radiotherapy to Intensity Modulated Radiotherapy (IMRT), resulting in better Organs at Risk (OAR) sparing and quality of life preservation [1]. IMRT planning intrinsically produces steep dose gradients, particularly at the border between target volumes (TV) and OAR. Control of patients’ immobilization and accurate volume definition of 3D images are of the utmost importance to ensure the improved therapeutic ratio of this technique. The segmentation of the various OAR is performed according to the anatomical knowledge. Gross Tumour Volume (GTV) is defined by anatomical modifications at clinical examination and on various imaging modalities. Prophylactic Clinical Target Volume (CTV) is selected and delineated according to universally accepted guidelines [2,3].

A tedious delineation is time-consuming, particularly in the complex head and neck region, where complete segmentation times of up to 180 minutes are reported, in contrast with the mere 20 minutes needed to create a simple 2D plan [4]. Delineation is also prone to large inter-observer variations for both OAR and CTV. In blind tests, parotids delineation on Computed Tomography (CT) or Magnetic Resonance Imaging (MRI) scans in 20 consecutive patients by three HNC radiation oncologists generated significant inter-observer variations with mean volume variations of about 50%, independent of the imaging modality [5]. In an international study on inter-clinician variability and its dosimetric impact, 32 different centers delineated the most common OAR on only one HNC patient CT set: brain, spinal cord, brainstem, both parotids and mandible. Significant variations were found for brainstem, both parotids and surprisingly spinal cord. After planning, this difference translated into significant variations in the irradiation of the so-called “reference segmentation”. There were differences reported of up to about 50% of the parotids D_mean_ and more than 20% of the brainstem D_max_[6]. In another monocenter study, 5 HNC experts performed a thorough qualitative analysis of the inter-observer variability after delineation of various OAR on 6 different CT sets. Despite the use of accepted delineation guidelines for both parotid and submandibular glands [7], significant differences persisted for mean volumes and concordance index. Geometrically, the largest differences (up to 3 mm) more often affected cranial and medial limits [8]. Finally, a multicenter survey providing a predefined GTV on the CT set of a given patient to 20 different HNC experts highlighted major differences in the CTV selection and delineation, dose prescription, chemotherapy prescription and Planning Target Volume (PTV) margin expansion [9]. The most striking finding was that, despite the common use of the international guidelines [2,3], neck node levels selection and delineation gave rise to large inter-observer variations of the low risk CTV volume (mean +/− SD = 205 +/− 123 cc). This is of particular concern since the compliance to radiotherapy guidelines is a documented prognostic factor for HNC treatments [10].

The concept of atlas-based automatic segmentation is appealing since it could help save significant delineation time while potentially reducing the inherent inter-observer variability. Different deformation registration strategies were developed, based on either individual patient data, averaged patient generation, multiple patient data [11] or, more recently, introduction of a volume post-processing by recognition of the key anatomical structures of the head and neck area [12,13].

An updated version of the automatic head and neck atlas was developed for an upcoming Brainlab treatment planning solution (Brainlab AG, Munich, Germany). It is based on the deformation registration of an atlas patient followed by active post-processing. The aim of the current study is to validate both time gain and accuracy of this software for the various OAR and CTVs that we use in clinical practice for unoperated HNC patients in two subpopulations: one “ideal” population (i.e. without any pathological node on at least one hemi-neck) and another “clinical” population (i.e. with a true clinical prescription according to the real N-status).

## **Methods**

### Patients and volumes selection

Two groups of un-operated HNC patients were defined, all referred to our Radiotherapy Department for definitive radiotherapy with or without concomitant chemotherapy. All patients were simulated head first supine with their head blocked by an anatomical neck cushion and an individual five-pin thermoplastic mask (Civco, The Netherlands). Without contra-indication, iodine contrast was injected in two phases: first 45 cc at 1.5 cc/sec, then after a rest of 120 sec by 40 cc at 2.0 cc/sec. CT acquisition started directly after the end of the second phase with a GE Lightspeed RT CT-scanner (General Electrics Healthcare, France); 2.5 mm thick slices were reconstructed from the upper orbita down to the lower part of the clavicula. Before processing, all data were imported into the Brainlab treatment planning software (iPlan RT 5.0 Beta, Brainlab AG, Germany) and anonymized with a number.

Group A. Ten patients (numbers 1 to 10) with at least one hemi-neck showing no pathological node (cN0). These patients were retrospectively selected from our database to test the algorithm without the interference of pathological nodes introducing a variation in the normal anatomy. All but one received iodine contrast injection. OAR selection was voluntarily restricted to spinal cord (SC), brainstem (BS) and homolateral parotid gland (PG). All possible neck node levels were individually selected in the cN0 hemi-neck: 1a, 1b, 2, 3, 4, 5, 6, retrostyloid (RST), retropharyngeal (RP) and retroclavicular (RCL).

Group B. Ten patients (numbers 11 to 20) with various N-stages to test the algorithm in real clinical conditions. To avoid any selection bias, these patients were the 10 first patients of another prospective study on adaptive radiotherapy (approved by the Ethics Committee of Clinique and Maternité Ste-Elisabeth on 28 September 2011 with National Belgian Reference Number B166201112118) proposed to patients on a consecutive scheme (i.e. without any other selection criteria than definitive radiotherapy and informed consent). All but one received iodine contrast injection. OAR were selected according to our clinical protocol: SC, BS, both PG, mandibula (M) and submandibular glands (SMG) only if level Ib was excluded from CTV. CTV selection (Additional file 1: Table S1) was clinically based on the one hand on primary tumor location and extension and on the other hand on N-stage, strictly according to the international guidelines [2,3]. All selected levels were fused together to form a unique “CTV 50 Gy” object for each patient.

### Atlas definition and registration algorithm

The reference atlas is built on the CT of a patient in standard position (head first supine with thermoplastic mask) with iodine contrast injection, from the vertex down to the 6^th^ thoracic vertebra. OAR and CTV nodal levels are individually segmented left and right. OAR list is extensive, regrouping bones (vertebrae, hyoid, clavicula), cartilages (cricoid, thyroid), muscles (sterno-cleido-mastoid), glands (PG, SMG, thyroid) and nervous structures (SC, BS, eyes, optic nerves, chiasm). Available nodal CTV levels are those described in the guidelines: 1a, 1b to 6, RST, RP and RCL. An independent expert who never worked with the first author performed all segmentations.

**Figure 1:**
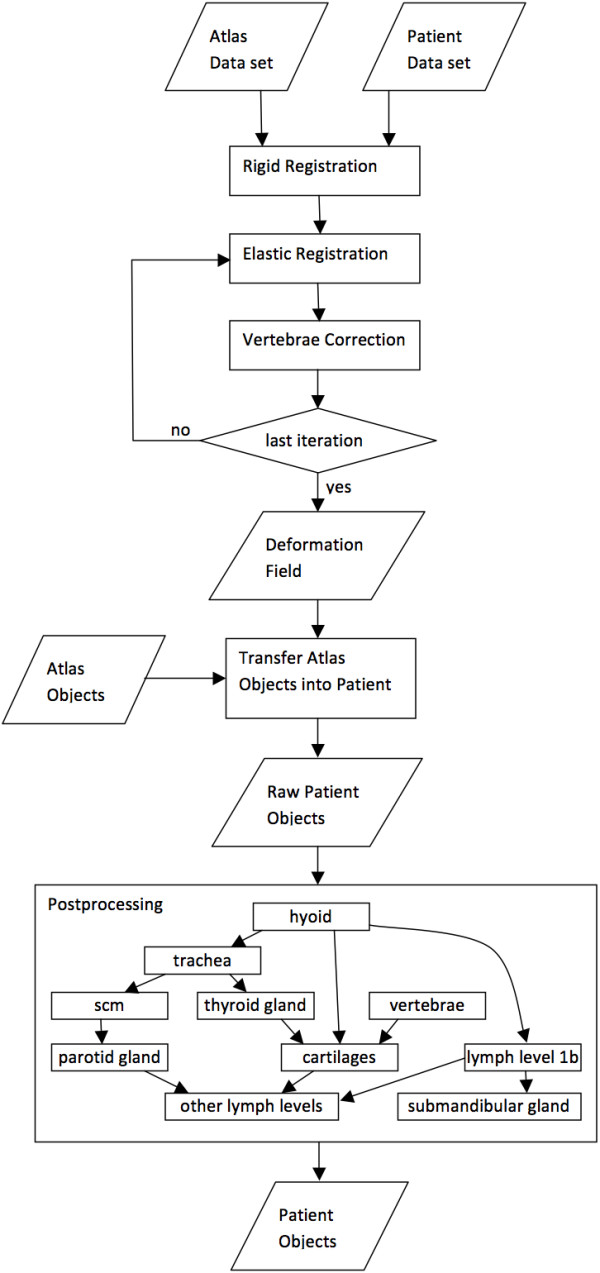
**Analytical diagram describing the workflow of the automatic segmentation algorithm.** SCM : sternocleidomastoid muscle.

Our approach entails a fully automatic segmentation algorithm using the most common atlas-based registration technique with various standard and model based support algorithms (Figure 1). It consists of two main parts. The first step is a standard atlas-based registration on CT data sets using intensity difference as similarity measure and starts with several rigid registrations. Standard elastic fusion algorithms often fail in matching the neck region due to the different relative orientations and positions between head and thorax and the curvature of the spine. The current elastic fusion algorithm is therefore restricted to preserve reasonable distances between the vertebras by transferring vertebra reference points from the atlas to the patient. The distances between these points are corrected to anatomically correct values by shifting the deformation vectors, which leads to a reliable stabilization of the neck region. Elastic fusion and stabilization are done in alternating order. Atlas registration is in any case a useful start point for segmentation and often already presents the correct point-to-point correspondence between atlas and patient data set. Since the neck region is of high variability a second post-processing step is necessary for segmentation. Furthermore a simple deformation and transfer of the atlas objects into the patient data set would break the Grégoire rules by violating plane-conservation [2,3]. The post-processing task of the current algorithm must therefore meet the high variability of the neck as well as the outlining guidelines. The lymph system of both sides is defined in the atlas as masks surrounding the actual lymph levels. The muscles and cartilages of the neck region are masks as well. All these masks are transferred to the patient and step by step post-processed using typical Hounsfield values and morphological operations to smooth the structures. First, thyroid gland and cartilages are segmented. Together with the bone reference points and the muscles they define the borders of the lymph levels, which are cut according to the delineation rules and define the final structures. It is implemented with standard image processing techniques based on typical Hounsfield values, size and shape of the neck structures. Alternating segmentation and reference point detection on bones, cartilages, muscles and lymph levels are generating the final structures.

### Segmentation

First author performed all segmentations. Reference segmentation was first performed manually (MAN volumes) and slice-by-slice for each patient. A few days later, MAN volumes were blinded and atlas-based automatic segmentation was applied (AUTO volumes). Last, AUTO volumes corrected if necessary (CORR volumes).

### Comparison pairs, metrics and statistics

Generated volumes were compared to each other in two ways. The first one was a comparison between AUTO and MAN volumes to measure the accuracy of the atlas-based segmentation. Secondly, a comparison between CORR and MAN volumes allowed measuring both the time needed to improve AUTO volumes and the intra-observer variability.

The human time needed to perform manual segmentation and necessary corrections was recorded in minutes, as well as the computer time to generate AUTO volumes.

Dice Similarity Coefficient (DSC) and average and maximal surface distances (ASD and MSD, respectively) were used for comparisons. DSC is a statistical parameter investigating the intersection volume of two objects by normalizing the intersection volume to a value between 0 (no overlap) and 1 (perfect overlap) and is defined as DSC_A,B_ = (2 |A ∩ B|) / (|A| + |B|) [14]. ASD and MSD are geometrical parameters expressed in millimetres [15,16], smaller distances reflect a better overlap.

Statistical analyses of the paired comparisons were performed using a double-sided T-test in Excel for Mac 2008 (Microsoft, Richmond, USA) with level of significance set at 0.05.

## **Results**

### Ideal conditions: group A

For the “ideal” patients in group A (Additional file 2:Table S2), delineating the OAR and nodal levels one by one took on average 44.9 min, three quarter of this time being devoted to the nodal levels. The use of the atlas led to necessary corrections that on average took 28.5 min, representing a time gain of 37% (*P* < 0.05). This gain was relatively larger for OAR (4.5 instead of 11.2 min, - 60%) than for nodal levels (24.0 instead of 33.7 min, - 29%). Of note, CORR time was equivalent to MAN time for levels 1a, 1b, 4, RP and RST.

**Figure 2:**
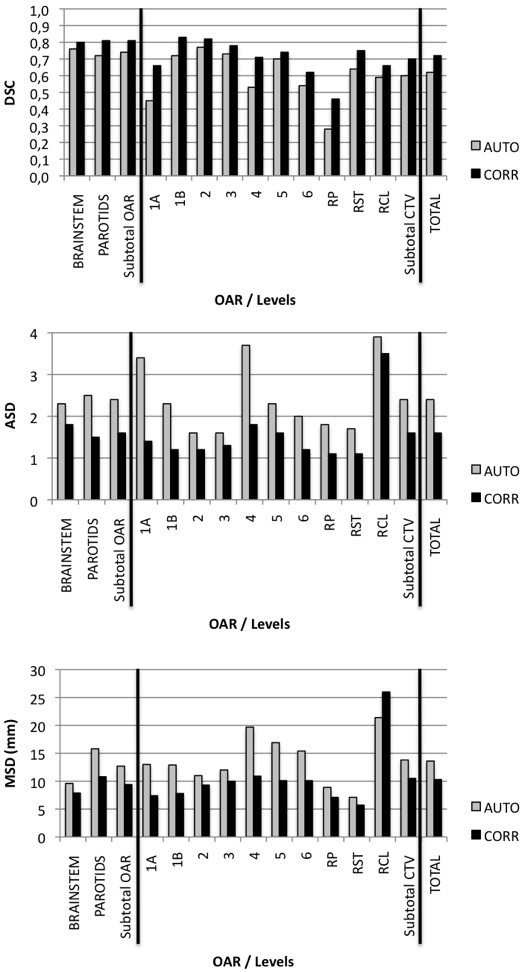
**Mean DSC, ASD and MSD in group A for automatic (AUTO) and corrected (CORR) volumes, both compared to manual (MAN) segmentation.** Vertical black line separates OAR section from CTV, and CTV from global mean (TOTAL). Last column of each section refers to mean values ("subtotal") for OAR and CTV. SC data were excluded since no correction was necessary. RP: retropharyngeal; RST: retrostyloid; RCL: retroclavicular.

**Figure 3:**
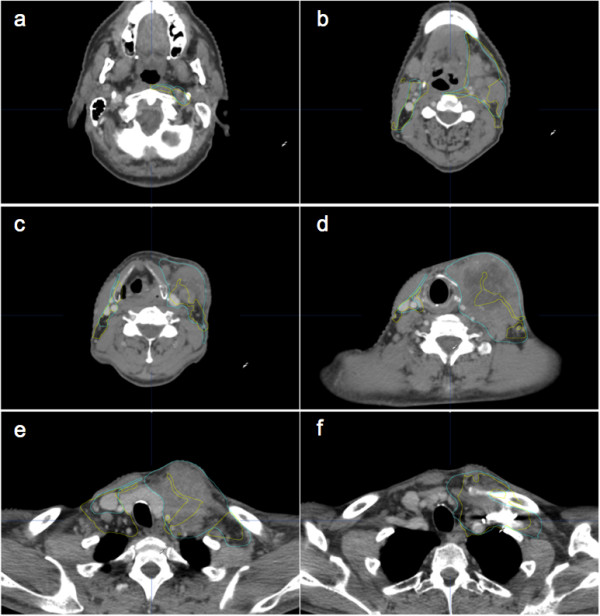
**Selected CT slices of patient 16 (N3-stage) with automatic (yellow lines) and manual (blue lines) segmentations.** Large normal anatomy distortions on the left side of the patient fool the algorithm that generates inaccurate automatic segmentation (images **c**, **d**, **e**). Levels depicted in the different panels: **a** = RS and RP left; **b** = 2 bilaterally, 1b and RP; **c** and **d** = 3 bilaterally; **e** = 4 bilaterally; **f** = RCL left.

**Figure 4:**
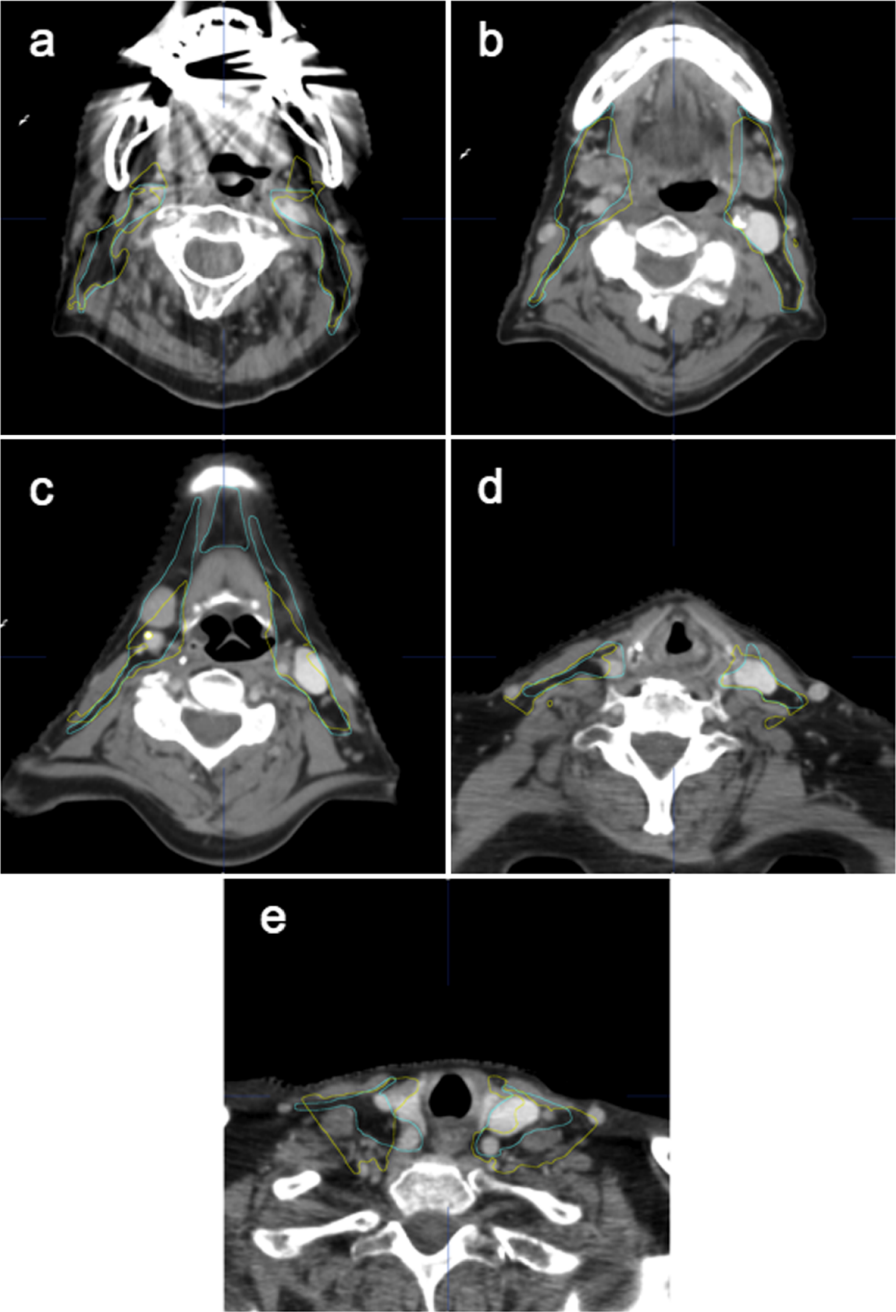
**Selected CT slices of patient 12 (N0-stage) with automatic (yellow lines) and manual (blue lines) segmentations.** Levels depicted bilaterally in the different panels: **a**= 2; **b** and **c** = 1b and 2; **d** = 3; **e** = 4.

Except for spinal cord, which showed minor deviations needing no further correction, AUTO volumes systematically needed some improvements, which are reflected by the better DSC, ASD and MSD parameters after correction (*P* < 0.05) (Figure 2). Levels 2, 3, 5, 6, RCL and OAR BS needed very few improvements, explaining also the large time gain for these volumes (Additional file 2: Table S2). As previously described, corrections for the other levels required more time. Level 1a is such a tiny volume that correcting even small deviations took as much time as drawing from scratch. For level 1b, most of the corrections were required at the cranial end, where the SMG is closely surrounded by muscles, making its differentiation difficult for the human eye as well as for the computer algorithm. Level 4 showed a systematic tendency to include scalenus muscles posteriorly (Figure 3e and Figure 4e), which consumed most of the correction time. For RP level, the algorithm showed difficulties to find the thin fatty layer anterior to the prevertebral muscle (Figure 3c). Most of the time, this level was wrongly located in the muscle itself, i.e. two to three millimetres posteriorly. The algorithm also tended to include the internal carotid artery laterally. Since this anatomical landmark intimately relates to RP and RST levels, correcting the lateral side of RP level automatically led to a necessary correction of the medial side of the RST level, explaining the large correction time needed for this last level. Lastly, it must be noticed that ASD and MSD were not improved by correcting the RCL level, reflecting its poorly defined anatomical boundaries and the inherent intra-observer variability.

### Clinical conditions: group B

**Figure 5:**
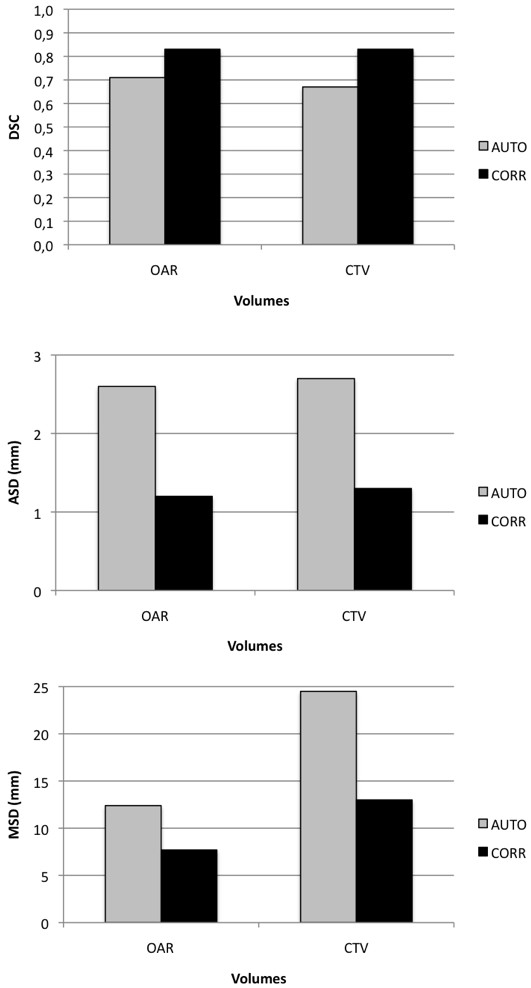
**Mean DSC, ASD and MSD in group B for automatic (AUTO) and corrected (CORR) volumes, both compared to manual segmentation.** OAR are grouped together (SC and M excluded).

On average, correction time after automatic segmentation was significantly shorter than manual segmentation (19.7 *vs* 34.5 min, *P* < 0.05). Like for group A, most of the gain was obtained for OAR (6.3 *vs* 16.4 min, *P* < 0.05). Again, SC did not need any correction, neither M except in one patient with teeth filling artefacts. Regarding the CTV, results are mixed, with patient 13 requiring an additional 2 min correction time, while 13 min were gained for patient 14. The average time gain for all patients in this series was 4.7 min. No significant time gain was observed for patients 13 (no iodine contrast injection), 16 (large N3) and 17 (large N2a) who demonstrated large anatomical deviations compared to the atlas (Figure 3). On the contrary, patients with few anatomic deviations were easy to correct since AUTO segmentation was fairly good (Figure 4). Again, statistically significant DSC, ASD and MSD parameters improvements were observed after correction of the automatically generated volumes (Figure 5). It underlines the necessary human supervision of the obtained result. It must be noted that SC and M structures were excluded from analysis since they practically did not need corrections.

### Computation time

Two different strategies were explored. In group A, automatic segmentation was performed through the iPlan® Net server (Brainlab AG, Germany). Time ranged from 25 to 50 sec, with an average of 39 sec. In group B, automatic segmentation was performed on a standalone workstation. For this group, time ranged from 90 to 155 sec, with an average of 116.5 sec.

## **Discussion**

The present study demonstrates the advantages and limitations of an automatic segmentation tool for OAR and nodal CTV to help radiation oncologists in their daily work, presently for the HNC treatments. It clearly helps saving time, particularly for the tedious OAR segmentation. We found a steady 40% reduction in both groups, independently of the number of delineated OAR. Though remaining significant, time gain was less prominent for the nodal CTVs, mainly because of individual anatomical variations. In the group without pathological node (group A), we could test the algorithm in ideal conditions and determine its limitations level by level. Some levels were easy to segment automatically and hence, needed very few corrections. This was particularly true for levels 2, 3, 5 and 6. On the other hand, levels 1a, 1b, RS and RP anatomical borders were more difficult to be determined automatically, mainly because of the lack of spontaneous contrast between the anatomical key structures (e.g. between the thin retropharyngeal fatty layer and the prevertebral muscles or between the summit of SMG and the surrounding muscles). Regarding level 4, a systematic inclusion of the scalenus muscles posteriorly relates to the lymph nodes mask that is voluntarily enlarged to take account of the matching uncertainties of the atlas. A tighter mask in this region could help solve the problem. Last, RCL level suffers large interpretations of its anatomical boundaries definition, resulting into large intra-observer variations, though delineation time can be saved. The study in true clinical conditions without patient recruitment bias (group B) also shed light on intrinsic limitations of automatic segmentation due to large deformations of normal anatomy or lack of iodine contrast injection. Finally and as a consequence of all these limitations, it was clearly demonstrated that -except for spinal cord and mandibula without teeth fillings- any automatically obtained volume should always be checked and corrected where necessary by an expert before planning.

Automatic segmentation accuracy is highly dependent on similarity between the underlying atlas and the patient [11]. Different strategies were recently explored in HNC patient population. The simplest atlas is based on an individual patient (IND) who must be chosen with care to ensure the similarity with the largest patients population. It is the least accurate strategy, particularly when dissimilarities arise, particularly in the node levels [17]. A more elegant way to overcome this limitation is to enlarge the atlas database by adding multiple patients; an averaged patient is created from the whole database and used afterwards as an atlas (AVG) [18-20]. Alternatively, multiple atlases generate multiple segmentations of the same object which, after combination, generate a single object (MUL) [17]. Anyway, none of these strategies include the recognition of the key anatomic structures boundaries that are used by the delineating physician [2,3]. This may potentially lead to violations of the cranio-caudal limits on the one hand [17] and to non compensation of large volumes overestimations on the other hand [18]. “Active Contour” (AC) or “Active Shape Modelling” post-processing after averaged atlas deformation constrains volumes within their anatomic boundaries, potentially compensating for these problems (AVG-AC) [12,13,21]. Our method takes the advantages of the AC methodology applied to the fast and simple IND atlas.

The comparison of all these different approaches to our one is limited by variations in the selected volumes (OAR only, CTV only or both), methodological differences and the multiple experts involved. Most groups reported their results by DSC metric, using the same methodology of MAN segmentation compared to AUTO, thus providing a reasonable basis for comparison. For BS, mean DSC of 0.83, 0.78, 0.58, 0.91 and 0.76 were observed for IND [17], MUL [15], AVG [19], AVG-AC [13] and our method, respectively. For PG, values of 0.80, 0.79, 0.67, 0.83 and 0.72 were calculated for the same methods, respectively. Regarding CTVs, the comparison is even more difficult since N-stage (most often N0) and selected levels were quite different from one study to another. Different publications reported mean DSC values of 0.60 (levels 2–4, IND) [17], 0.67 (levels 1–5, MUL) [15], 0.79 (levels 1–6 + RP, AVG) [20], 0.46 (levels 1–6, AVG-AC) [21], 0.70 (levels 2–4, AVG-AC) [12,13], 0.77 (level 1b, AVG-AC) [13] and 0.60 (all levels in group A, our method). To improve the comparison validity of our method, our mean DSC value rises up to 0.69 for levels 1b-4 only. Computation time varied also greatly: from 7 min [17,19] up to 21 min [12]. Our recorded times -less than 2.5 min on workstation and less than 1.5 min on the iPlan® Net server- compare favourably and are under the three minutes practical cut-off [22].

Whatever method used, none produces automatic volumes directly usable for planning. It was nicely demonstrated with a MUL algorithm applied to nine patients that significant underdosage is observed in reference PTV when planning is performed based on a PTV generated from automatic contours. Underdosage of 11 Gy may be observed even for very good metrics like DSC = 0.8 and ASD < 1 mm. Of note, this had no impact on OAR [23]. Corrections improve automatic segmentation both quantitatively (DSC increases by 0.1 on average [15,20] and qualitatively, but still take time. In our study, the time gain was about 40% compared to the manual segmentation time, which is in line with the 26 to 47% range reported in the literature and being an inverse function of the expertise of the physician [20,24]. The time savings hold only true for OAR and prophylactic CTV delineation, not for GTV and high dose CTV. Of course, one could argue that summing up the times needed to delineate all these volumes at once would result in only a small overall time saving. For us it is important to save time on tasks of less concern and use these gained resources for more important challenges, e.g. reviewing a medical file and delineating a GTV extension.

Using an atlas may also improve inter- and intra-observer variability. In a study where five different HNC radiation oncologists each delineated the CTV for five patients, it was convincingly demonstrated that the corrected atlas-based contours showed less inter-observer variability than manual segmentations. Corrected contours were on average larger than the manual ones, highlighting the potential bias induced by automatic contours [20]. The same trend was also detected for OAR delineation when two different experts teams on two different populations used two different strategies. In population 1 with team 1, BS, PG and M were first segmented manually then segmented automatically by atlas. In population 2 with team 2, these OAR were segmented first automatically, then manually corrected. In population 2, mean volumes were significantly less different than for population 1. DSC and specificity were on average 20% better in population 2 than in population 1 [19]. In our study, the imperfect matching between corrected volumes and manual ones reflects the bias induced by the automatic delineation. However, both volumes gave satisfaction from visual viewpoint and also reflect the best intra-observer variability that could be achieved.

Last, some points were not directly addressed and could be further studied.

● Inter-observer variability could be checked on our patients by repeating the study on a blinded multicentre frame.

● All our patients were unoperated; the algorithm may be evaluated against a post-operative population, which has never been tested. However, since anatomy would even be more deformed than in our population B, we think that more “intelligent” algorithms should be developed to overcome this limitation.

● Some more OAR could be included in the atlas (e.g. swallowing structures) and make the purpose of a new validation. Anyway, since different guidelines are available [25-27], one must wait for a global consensus to avoid potential criticisms about a chosen method.

## **Conclusion**

An updated commercial HNC atlas was validated for both OAR and prophylactic CTV delineation, proving its interest as help in daily clinical work. It was clearly demonstrated as time-saving, even though a physician should always review generated contours. Potential small improvements were highlighted, e.g. use of MUL atlas, improved correction tools or introduction of new OAR in the atlas (e.g. swallowing structures [25-27]).

### Consent

Written informed consent was obtained from the patient for the publication of this report and any accompanying images.

## Abbreviations

2D: Two-dimensional; AC: Active contour; ASD: Average Surface Distance; AUTO: Automatic; AVG: Averaged; BS: Brainstem; CORR: Corrected; CT: Computed tomography; CTV: Clinical target volume; DSC: Dice Similarity Coefficient; GTV: Gross Tumour Volume; HNC: Head and neck cancer; HP: Hypopharynx; IMRT: Intensity modulated radiotherapy; IND: Individual; L: Left; M: Mandibula; MAN: Manual; MRI: Magnetic Resonance Imaging; MSD: Maximal surface distance; MUL: Multiple; OAR: Organs at risk; OC: Oral cavity; OP: Oropharynx; PG: Parotid gland; PTV: Planning Target Volume; R: Right; RCL: Retroclavicular; RP: Retropharyngeal; RST: Retrostyloid; SC: Spinal cord; SMG: Submandibular gland; TV: Target volume.

## Competing interests

Financial: JFD received speaker fees from Brainlab; AB is a Brainlab employee; Radiotherapy Department received free software as compensation.

Non-financial: none to declare.

## Authors’ contributions

JFD designed the study, acquired the data, performed the analyses and wrote most of the article. AB developed and designed the software and wrote the technical part. All authors read and approved the final manuscript.

## Authors’ information

JFD was PhD fellow (1999 – 2002) and clinical fellow (2002–2003 and 2005–2006) of Professor Vincent Grégoire in Université Catholique de Louvain, main author of the international guidelines [2,3] . Since 2006, works as Full Time Board Certified Radiation Oncologist in Clinique & Maternité Ste-Elisabeth in Namur. 40% of activity relates to HNC patients treatment, with on average two new HNC patients a week. 20% of activity devoted to imaging and radiation oncology translational research in the frame of the academic structure NAmur Research Institute for Life Sciences (NARILIS).

## Supplementary Material

Additional file 1: Table S1 Nodal levels selection for CTV 50 Gy of patients in group B. Click here for file

Additional file 2: Table S2 Average segmentation times in group A. Click here for file

## Meeting presentation

As a poster, 31^st^ ESTRO conference, 9–13 May 2012, Barcelona (Spain).
