# Supplementary material for: Atlas-based automatic segmentation of head and neck organs at risk and nodal target volumes: a clinical validation
Source: Radiat Oncol. 2013 Jun 26;8:154. doi: 10.1186/1748-717X-8-154 (PMC3722083; doi:10.1186/1748-717X-8-154)
Supplement: Additional file 2: Table S2 — Average segmentation times in group A. [file 1748-717X-8-154-S2.pdf]

OAR / LEVEL	TIME (min)	
	MAN	CORR
BRAINSTEM	2,90	1,20
SPINAL CORD	3,90	0,00
PAROTIDS	4,40	3,30
Subtotal OARs	*11,20*	*4,50*
1A	1,10	1,00
1B	3,90	3,70
2	5,30	2,40
3	3,70	2,30
4	2,80	3,00
5	2,90	1,60
6	6,60	3,10
RP	2,50	1,70
RST	1,20	1,50
RCL	3,70	3,70
Subtotal CTV	*33,70*	*24,00*
TOTAL	*44,90*	*28,50*
